# Positron Emission Tomography/Computed Tomography Scanner for Pre-Transcatheter Aortic Valve Replacement Assessment: Rethinking Our Resources

**DOI:** 10.1016/j.jaccas.2026.108459

**Published:** 2026-05-22

**Authors:** Giulia Passaniti, Roosha Parikh, George Petrossian, Newell Robinson, Jaffar Khan, Omar K. Khalique

**Affiliations:** a Division of Cardiovascular Imaging, St Francis Hospital and Heart Center, Roslyn, New York, USA; b Heart Valve Center, St Francis Hospital and Heart Center, Roslyn, New York, USA

**Keywords:** aortic valve, computed tomography, imaging

## Abstract

**Background:**

Since the first transcatheter aortic valve replacement (TAVR), there has been an exponential increase in the number of patients diagnosed and treated for aortic stenosis.

**Case Summary:**

A 79-year-old man, with severe symptomatic aortic stenosis and a past medical history of coronary artery bypass in 2006 and percutaneous coronary stent implantation in 2016, presented to our Heart Valve Center for pre-TAVR assessment. Preprocedural computed tomography angiography was completed on a positron emission tomography (PET)/computed tomography (CT) machine. The patient underwent successful implantation of TAVR, with no evidence of leak on postoperative echocardiographic assessment on days 1 and 30.

**Discussion:**

Cardiac computed tomography angiography is the established gold standard for pre-TAVR evaluation in patients with no absolute contraindications; we may soon face a resource saturation problem. Therefore, we should aim to use our resources wisely.

**Novelty:**

A possible solution to the issue of CT scanner saturation lies in using our resources more efficiently: for example, pre-TAVR planning could be performed on PET/CT scanners, providing comparable results.

**Take-Home Message:**

Pre-TAVR assessment can be safely performed on a PET/CT machine.

## Visual Summary

**Figure:**
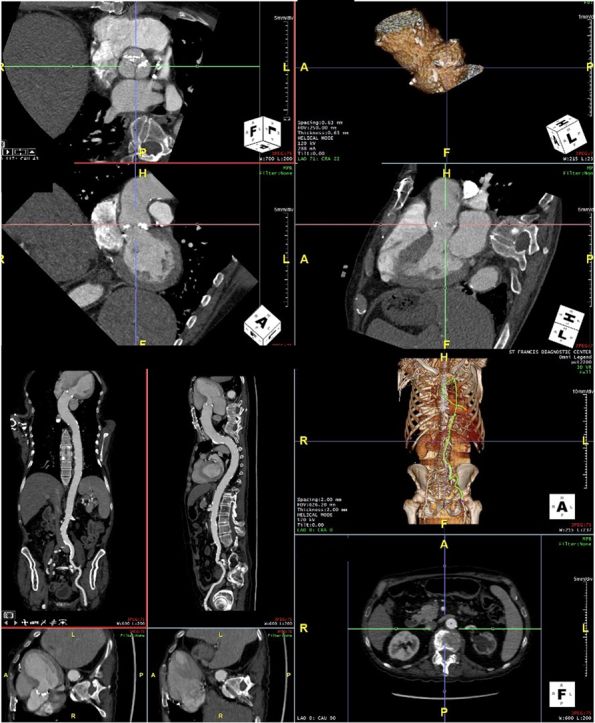

Since the implantation of the first transcatheter aortic valve prosthesis performed in 2002, advancements in diagnostic and therapeutic tools have led to an exponential increase in the number of patients diagnosed and consequentially treated for severe symptomatic aortic stenosis.

Valvular guidelines have progressively broadened the spectrum of patients who can benefit from transcatheter aortic valve replacement (TAVR) and, as the EARLY TAVR trial has recently demonstrated, treating patients with asymptomatic severe aortic stenosis reduces the composite risk of death, stroke, and unplanned cardiovascular hospitalization, leading to a class IIa indication for patients with preserved ejection fraction (≥50%), according to the latest European Society of Cardiology guidelines.^1^^,^^2^

In our everyday practice, these will directly increase the number of TAVR performed and, accordingly, the number of diagnostic examinations required for both procedural planning and follow-up: transthoracic and transesophageal echocardiogram, cardiac computed tomography angiography (CTA), and cardiac catheterization.

Cardiac CTA plays a critical and increasingly prominent role in various cardiac conditions, included but not limited to the preplanning of structural procedures, diagnosis and follow-up of coronary artery disease, assessment of bypass graft/stent patency, preplanning for electrophysiology procedures, and assessment of congenital heart disease.^3^ In addition, most centers share computed tomography (CT) scanners with a general radiology service.

Therefore, we are currently facing a system saturation of CT scanners, resulting in longer waiting times and potential repercussions on patients’ health.

Thus, it is critical to use all resources available to increase access and efficiency. One example is the use of a dual-purpose scanner such as positron emission tomography (PET)/CT for pre-TAVR planning.

PET/CT combines 2 different modalities in a single scanner: this scanner can be used for dedicated PET nuclear imaging, which provides functional and metabolic data based on the uptake of specific radiotracers, or for an isolated CT function, which provides high-resolution structural and anatomical information.

## Case Summary

The patient is a 79-year-old man, with severe, symptomatic aortic stenosis and a past medical history of coronary artery bypass in 2006, percutaneous coronary stent implantation in 2016, anemia, and benign prostate hyperplasia. The patient was referred to a new imaging center located in the same building as the Heart Valve Center office with lower backlogs than dedicated CT scanners in the same hospital system, in order to avoid delays in care.

**Figure 1:**
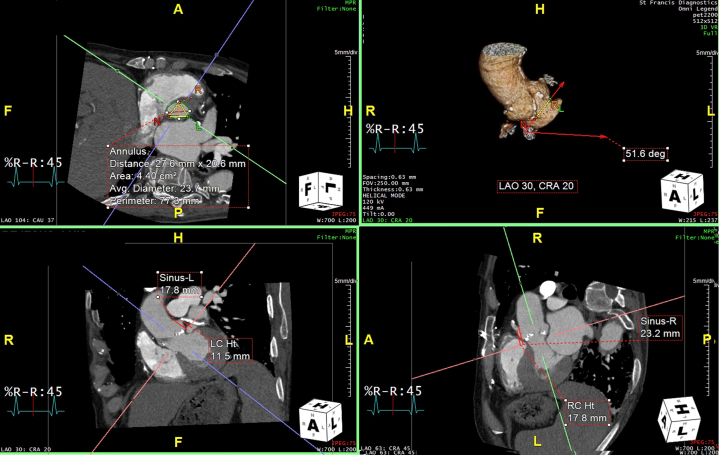
Double Oblique Multiplanar Reconstruction and 3-Dimensional Volume Rendering Showing Calcific Aortic Stenosis In late systole (45% phase), the aortic annulus (minimum and maximum diameters, area, and perimeter) and the left and right coronary heights and the sinuses of Valsalva heights were measured.

**Figure 2:**
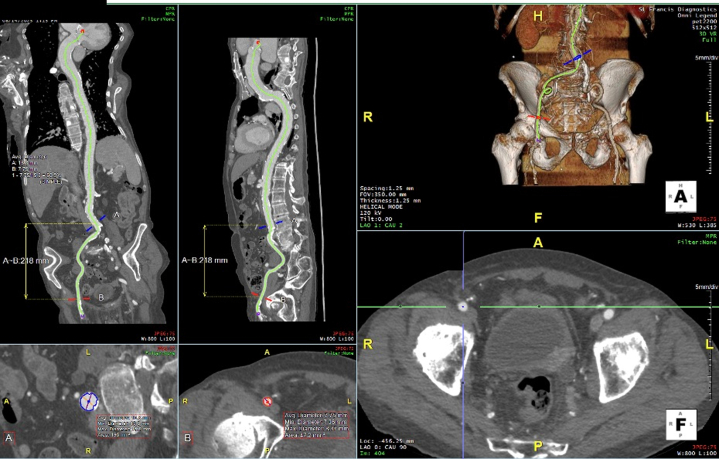
Coronal, Sagittal, and Axial Views Obtained for the Evaluation of Peripheral Access A non–electrocardiography-gated aortogram scan was performed, extending from the aortic arch cranially to the femoral arteries caudally. Curved multiplanar reconstruction reconstruction allowed evaluation of the entire thoracoabdominal aorta. Image quality was deemed excellent.

Preprocedural assessment was performed by a GE Discovery 128-slice PET/CT 710 scanner: the retrospective electrocardiography (ECG)-gated volume acquisition was acquired using the dedicated CT function of the scanner with a tube potential of 120 kV and a tube current of 288 mA. At the time of the scan, the patient was in normal sinus rhythm, with a heart rate of 67 beats/min. Intravenous administration of 95 mL of low-osmolar contrast material (Omnipaque 350; GE HealthCare) was performed via an antecubital vein, at a rate of 4.5 mL/s, using a bolus-tracking technique with the region of interest placed in the thoracic descending aorta. The scan was automatically triggered when the threshold of 100 Hounsfield units was reached. Image quality was excellent, and preprocedural measurements were performed in the systolic phase (Figure 1). Non–ECG-gated CTA of the neck, thorax, abdomen, and pelvis was performed with a slice thickness of 0.625 mm (Figure 2), in order to assess proper vascular access site and feasibility, tortuosity, minimal diameter, and so on.

**Figure 3:**
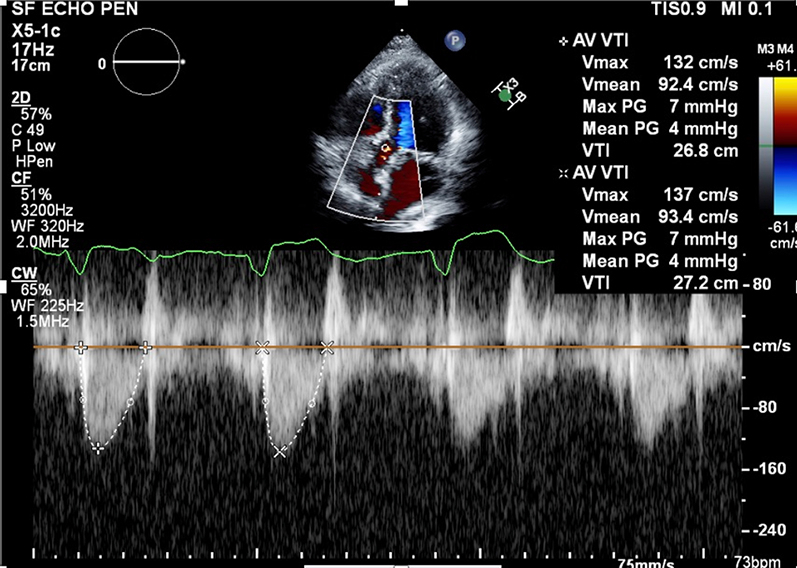
Postprocedural Transthoracic Echocardiogram Assessment CW Doppler across the aortic prosthesis shows normal transvalvular gradients (peak gradient 7 mm Hg, mean gradient 4 mm Hg, V_max_ 137 cm/s). CW = continuous wave Doppler.

The patient underwent TAVR via transfemoral access with 26-mm Edwards Sapien 3 valve, with no intraprocedural complications. A postoperative transthoracic echocardiogram at 1 and 30 days showed normal transvalvular gradients (Figure 3) and no paravalvular or intravalvular leaks (Video 1).

To the best of our knowledge, this is the first reported experience of pre-TAVR assessment on a PET-CT scanner.

## Novelty of the Submission

Our case demonstrates that a PET/CT scanner can be effectively used to assess the feasibility of TAVR during preprocedural evaluation, with favorable outcomes for the patient. To the best of our knowledge, this is the first report of the dedicated CT function of a PET-CT scanner being used for acquisition of imaging for preprocedural structural heart planning. Image quality of both the ECG-gated cardiac acquisition and the non–ECG-gated aortogram was unanimously assessed as excellent (5) by 2 experienced cardiac imagers and 1 experienced radiologist, based on a 5-point Likert scale (1, unacceptable image quality, 2, poor image quality; 3, acceptable image quality; 4, good image quality; 5, excellent image quality). This resulted in a well-expanded and properly seated prosthesis, with normal transvalvular gradients and no paravalvular or intravalvular leaks.

## Future Directions

Future case reports and research will help confirm and expand the validity of use of multimodality/multiuse scanners such as PET-CT for flexible structural heart preprocedural planning.

## Conclusions

As our case illustrates, hybrid scanners can deliver high diagnostic-quality images for preprocedural planning of TAVR. Whether the image quality obtained with a PET/CT scanner can be used to plan other structural interventions is a subject of future research. Becoming more efficient with resource utilization, including multiuse scanners such as PET/CT, is critical to improve throughput and expedite patient access.

**Visual Summary:**
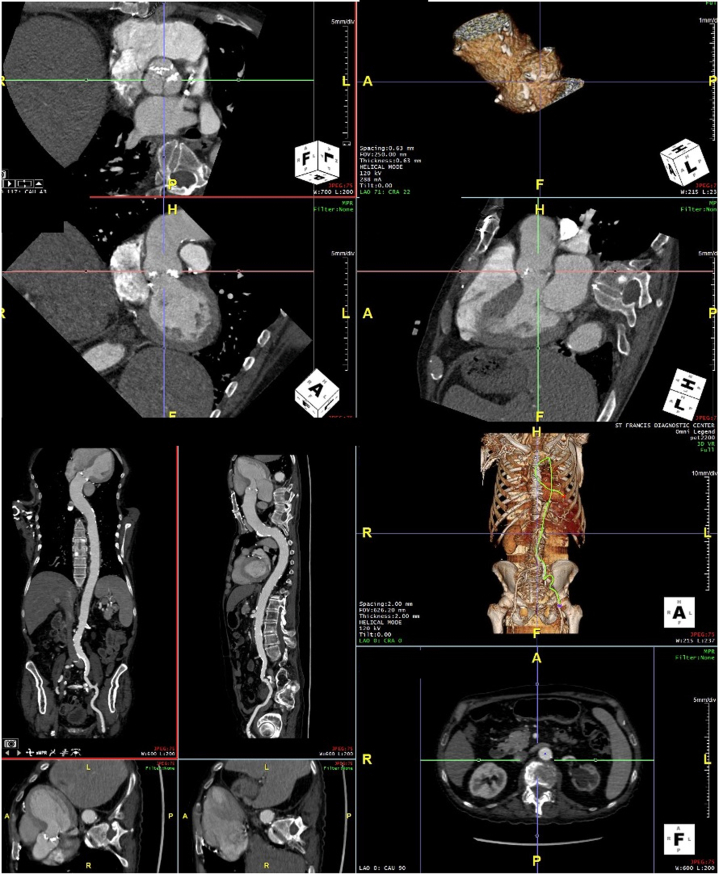
Comprehensive Pre-TAVR Assessment Using PET/CT Scanner The PET/CT scanner offers a valid and safe alternative to a dedicated CT system, providing high-quality images suitable for a comprehensive and thorough pre-TAVR assessment. Retrospective ECG-gated volume acquisition was acquired, and double oblique multiplanar reconstruction (MPR) and 3-dimensional volume rendering showed calcific aortic stenosis. Non–ECG-gated aortogram scan was acquired, extending from the aortic arch cranially to the femoral arteries caudally. Curved MPR reconstruction allowed evaluation of the entire thoracoabdominal aorta. Image quality was deemed excellent. CT = computed tomography; ECG = electrocardiography; PET = positron emission tomography; TAVR = transcatheter aortic valve replacement.

## Funding Support and Author Disclosures

Dr Khalique has served as a consultant for Edwards, Siemens, Philips, and Abbott. Dr Parikh has served as a consultant to HeartFlow and Cleerly. Dr Khan received consulting/proctoring fees from Abbott, Edwards Lifesciences, and Medtronic; has equity in Transmural systems and Cuspa Medical; and is coinventor on patents, assigned to the National Institutes of Health, on leaflet modification devices. All other authors have reported that they have no relationships relevant to the contents of this paper to disclose.

**Take-Home Messages**

• The positron emission tomography/computed tomography scanner offers a valid and safe alternative to a dedicated computed tomography system, providing high-quality images suitable for a comprehensive and thorough pre–transcatheter aortic valve replacement assessment.
• The electrocardiography-gated volume acquisition allows accurate measurement of the aortic annulus, coronary heights, and leaflet lengths to determine the most appropriate prosthesis type and size for each patient.
• The non–electrocardiography-gated computed tomography angiography enables evaluation of the size, tortuosity, and calcification of the thoracoabdominal aorta and femoral arteries, facilitating optimal vascular access planning.

## Appendix

Video 1 1-day post op TTE: 5-chamber and 3-chamber apical view, with and without color show no intra-valvular or peri-valvular leaks. TTE = transthoracic echocardiogram. Five-chamber and 3-chamber apical views with color-compare show no intravalvular or perivalvular leaks.
